# Ultra-long-TE arterial spin labeling reveals rapid and brain-wide blood-to-CSF water transport in humans

**DOI:** 10.1016/j.neuroimage.2021.118755

**Published:** 2021-11-24

**Authors:** Leonie Petitclerc, Lydiane Hirschler, Jack A. Wells, David L. Thomas, Marianne A.A. van Walderveen, Mark A. van Buchem, Matthias J.P. van Osch

**Affiliations:** a C.J. Gorter Center for High Field MRI, Department of Radiology, Leiden University Medical Center, Leiden, Netherlands; b Leiden Institute for Brain and Cognition (LIBC), Leiden, Netherlands; c Department of Radiology, Leiden University Medical Center, Leiden, Netherlands; d UCL Centre for Advanced Biomedical Imaging, Division of Medicine, University College London, London, United Kingdom; e Neuroradiological Academic Unit, Department of Brain Repair and Rehabilitation, UCL Queen Square Institute of Neurology, University College London, London, United Kingdom; f Dementia Research Centre, UCL Queen Square Institute of Neurology, University College London, London, United Kingdom; g Wellcome Centre for Human Neuroimaging, UCL Queen Square Institute of Neurology, University College London, London, United Kingdom

**Keywords:** Arterial spin labeling, Blood-csf barrier, Neurofluids, Glymphatics, Brain clearance, Water transport, Dynamic compartmental modeling

## Abstract

The study of brain clearance mechanisms is an active area of research. While we know that the cerebrospinal fluid (CSF) plays a central role in one of the main existing clearance pathways, the exact processes for the secretion of CSF and the removal of waste products from tissue are under debate. CSF is thought to be created by the exchange of water and ions from the blood, which is believed to mainly occur in the choroid plexus. This exchange has not been thoroughly studied in vivo.

We propose a modified arterial spin labeling (ASL) MRI sequence and image analysis to track blood water as it is transported to the CSF, and to characterize its exchange from blood to CSF. We acquired six pseudo-continuous ASL sequences with varying labeling duration (LD) and post-labeling delay (PLD) and a segmented 3D-GRASE readout with a long echo train (8 echo times (TE)) which allowed separation of the very long-T_2_ CSF signal. ASL signal was observed at long TEs (793 ms and higher), indicating presence of labeled water transported from blood to CSF. This signal appeared both in the CSF proximal to the choroid plexus and in the subarachnoid space surrounding the cortex. ASL signal was separated into its blood, gray matter and CSF components by fitting a triexponential function with T_2_s taken from literature. A two-compartment dynamic model was introduced to describe the exchange of water through time and TE. From this, a water exchange time from the blood to the CSF (T_bl->CSF_) was mapped, with an order of magnitude of approximately 60 s.

## 1 Introduction

Brain waste clearance mechanisms are still poorly understood, standing in stark contrast to our much deeper general understanding of brain metabolism and function. The interaction of cerebrospinal fluid (CSF) and interstitial fluid (ISF) is thought to form a key aspect of clearance; however, the exact circulating pathways of these fluids, and the driving forces for motion in and out of tissue, are still under debate. In fact, this field of study, also sometimes referred to as “glymphatics” after one of the hypothesized pathways for these processes (Iliff et al., 2012), has garnered much attention and excitement in recent years as our understanding of brain clearance (Albargothy et al., 2018; Liu et al., 2020;Mestre et al., 2018;Mestre et al., 2020;Wardlaw et al., 2020;Xie et al., 2013;Fultz et al., 2019 van Veluw et al., 2020;Nedergaard and Goldman, 2020;Tarasoff-Conway et al., 2015) has begun to improve. One key component in solving the brain clearance puzzle is to obtain a complete picture of CSF circulation, including the spatial location of exchange sites between the blood and CSF. Most of the CSF production, i.e. net flow of water from blood into CSF compartments, is thought to take place at the blood-CSF barrier (BCSFB) in the choroid plexuses situated in the ventricles. However, there is mounting evidence that this is not the sole site of CSF production, and other sources have been proposed, but not yet identified in vivo (Hladky and Barrand, 2016; Orešković et al., 2017; Mehemed et al., 2014). Since waste clearance is an essential component of healthy brain function, and disruptions in this process may lead to the accumulation of molecules associated with neu- rodegenerative diseases (Tarasoff-Conway et al., 2015), a non-invasive method to measure and characterize blood-to-CSF water exchange is much needed. Moreover, it is essential to move towards human, in vivo experiments, to build upon current knowledge which has come predominantly from rodent experiments, without direct replication in humans.

As CSF is a fluid comprised mostly of water, the main factor contributing to CSF production is the net transport of water across the BCSFB. Arterial spin labeling (ASL) can be exploited to measure the delivery of labeled blood water across the BCSFB, as it allows monitoring of water entering the brain after it has been magnetically labeled inside the large arteries of the neck. In traditional ASL sequences, there is a short wait (post-labeling delay, PLD) between labeling and imaging to allow the bolus of label to reach the tissue compartment. The passage of water across the BCSFB is thought to happen on a slower and smaller scale than gray matter perfusion, and therefore there is typically little to no signal detected in CSF with ASL. However, by prolonging the labeling duration and using a longer PLD, ASL signal should cross the BCSFB and accumulate in the CSF at a measurable level, which is supported by the very slow T_1_ decay of CSF (T_1CSF_ ~4300 ms), thereby allowing a build-up of labeled signal over time (Zhao et al., 2020). Additionally, the long T_2_ (>1500 ms at 3T (Spijkerman et al., 2018)) of CSF can be leveraged to isolate the signal coming from this compartment from perfusion signal within the brain tissue by using a long echo time, as has been shown previously in mice (Evans et al., 2020). Imaging at multiple time-points and with a long echo train can allow dynamic compartmental modeling to characterize the temporal dynamics of water exchange between blood and CSF.

This study aims to use a multi-time-point, multi-echo ASL protocol to measure the amount and location of water transport across the BCSFB in the human brain, and to model and characterize the dynamics of blood-CSF water exchange.

## 2 Materials and Methods

### 2.1 Acquisition

All data were acquired on a Philips Achieva XT 3T system (Philips, Best, the Netherlands) with a 32-channel head coil. Two protocols were used in this study: the main protocol (henceforth referred to as the “multi-PLD” protocol) aimed to dynamically follow labeled blood water from its arrival in the imaging region and subsequently through to its transport across the blood-CSF barrier. The second protocol was for validation purposes, acquired on a separate day, to assess reproducibility of the method, and included a scan with labeling above the imaging region to confirm that the observed signal did indeed originate from labeled arterial blood. 12 healthy subjects (3 male, 9 female, ages 24–66) underwent the multi-PLD protocol and, of those, 4 (1 male, 3 female, ages 30–64) were also scanned with the validation protocol on a separate day. All subjects provided written informed consent in accordance with our center’s IRB regulations.

**Fig. 1:**
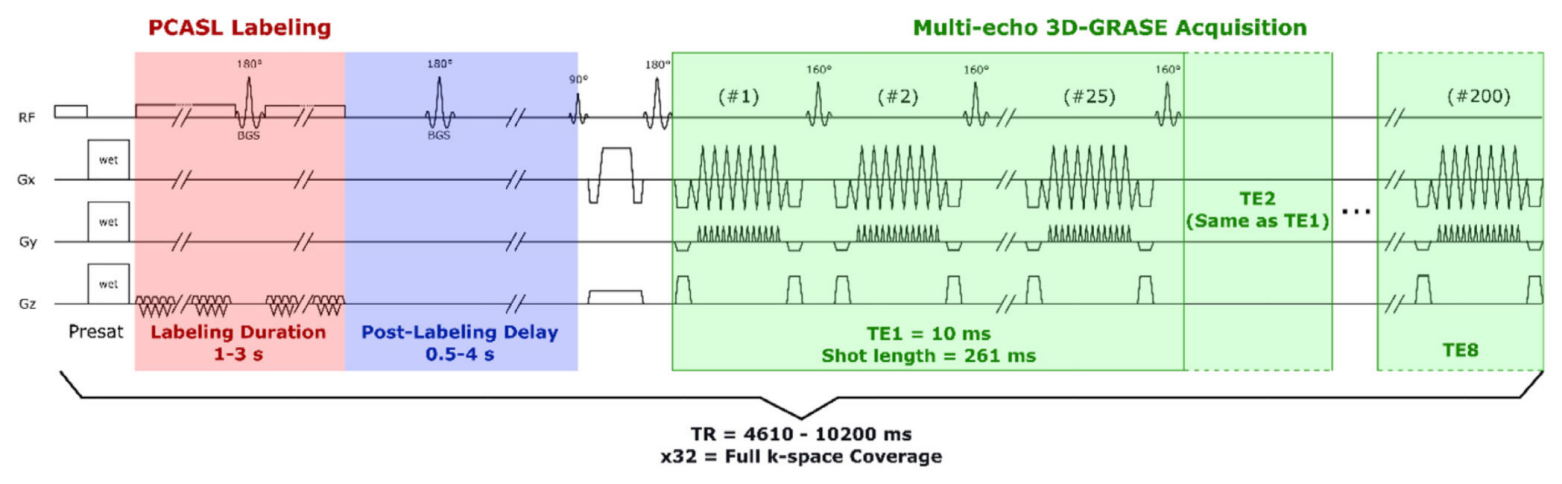
Diagram of the multi-echo ASL sequence used in the main protocol of this study. Timings and gradient strengths are not drawn to scale.

The multi-PLD protocol (shown schematically in Fig. 1) consisted of 6 pseudo-continuous ASL (PCASL) scans with varying labeling duration (LD = 1, 1, 1.5, 2, 3, 3 s) and post-labeling delay (respectively, PLD = 0.5, 1, 1.5, 2, 2.5, 4 s), in addition to a M_0_ scan without labeling. For all of these, a segmented 3D-gradient and spin echo (3D-GRASE) readout was employed with multiple echo times, acquired by extending and dividing the turbo-spin echo (TSE) train into 8 sections, corresponding to 8 echo times (TE =10 + n^*^261 ms, *n =* 0:7 and TE =9.7 + n^*^243, *n =* 0:7 for ASL and M_0_ scans, respectively). The readout was segmented into 4 separate acquisitions in the EPI phase encoding direction (anterior-posterior in this case). The acquisition matrix was 64 ×60 (RLxAP) and the imaging volume was reconstructed to a matrix of 80 × 80 voxels and 28 slices (resolution 3 × 3 × 6 mm^3^). This was accomplished with a TSE factor of 200 (i.e. 200 refocusing pulses, the first one with a flip angle of 180°, and 160° for all subsequent refocusing pulses), where the echo train is divided into 8 echoes (25 refocusing pulses per echo, per acquisition), a slice oversampling factor 1.8, and an EPI echo train length of 15. In this implementation, the acquisition is repeated four times and averaged before reconstruction. The pCASL scans had variable TR in order to accommodate the total length of the LD/PLD (TR = 4610, 5200, 6200, 7200, 8700, 10,200 ms respectively) and the M_0_ scan had a TR of 10 s to ensure full recovery of magnetization of the blood and tissue, and almost full recovery of CSF. The PCASL scans also employed two background suppression pulses with timings optimized to reduce signal from both static tissue and CSF. The total time of this protocol including all scans was approximately one hour.

The validation protocol was acquired in a separate session on a different day and included a repeat of the PCASL scan with LD/PLD = 3/4 s, the same scan with the labeling plane placed symmetrically above the brain, and a high resolution 3D-FFE T_1_-weighted image (TR/TE 9.8/4.6 ms, resolution 0.875 × 0.875 × 1.2 mm^3^ (APxRLxFH)). This session also included additional background suppression experiments (not discussed here) and the total acquisition time was just under one hour.

Finally, multi-echo spin-echo sequences are known to create stimulated echoes which can affect T_2_ quantification (or separation of compartments based on T_2_ in this study). Due to the high number of refocusing pulses, in combination with the lower flip angle (160°) of all but the first refocusing pulse of our sequence, it was important to investigate this effect on our measurements. In one subject (#9), we performed an additional scan session in which we repeated the multi-echo scan with LD/PLD 3/2.5 s, as well as acquiring two scans employing a T_2_-prep module (single echo, with 16 adiabatic refocusing pulses) to reproduce the third and fourth echo times (TE = 532, 793 ms), in order to compare the observed signals. For this experiment, background suppression pulse timings had to be changed to null the signal at the beginning of the T_2_-prep module rather than at the beginning of readout, so as to not interfere with the T_2_-prep module.

### 2.2 Validation and image pre-processing

ASL control and label images were pair-wise subtracted to extract the ASL signal at all PLDs and TEs. A brain mask was created to remove any signal from the background by thresholding the first-echo M_0_ image. Similarly, a manual threshold was applied to the first TE image of the (LD = 2 s, PLD = 2 s) ASL signal to obtain a gray matter mask (the threshold was chosen by an operator by visual inspection of the resulting mask to preserve the anatomical features of gray matter), and to the M_0_ scan at TE = 498 ms to create a CSF mask. To avoid overlap, all voxels that were assigned as being in both the CSF and GM masks were removed from the GM mask. To assess the reproducibility of the method and presence of artefactual signal, the signal from the (LD = 3 s, PLD = 4 s) scan acquired with (i) the multi-PLD protocol; (ii) the validation protocol; and (iii) the labeling plane above the brain (which was acquired with the same parameters except for the label position) were compared. The methods for reproducibility analysis in ASL traditionally use the different signal averages acquired in one sequence to compare variability within and between sequences. (Gevers et al., 2011; Mutsaerts et al., 2014) However, because of the nature of our multi-echo sequence, signal accumulation occurs by additional oversampling and averaging of k-space in the z-direction, resulting in only a single image per PLD and TE combination. Standard reproducibility methods were therefore not available to us for this analysis. Instead, we compared the subtracted smoothed ASL signal between the multi-PLD and validation scans (after coregistration in SPM12 (London, UK) and normalized by the average M_0_ signal for each echo time) and compared this to the signal obtained when labeling above the head. This allows us to observe the amount by which signal varies between scans and the spatial distribution of these variations.

Prior to performing voxel-wise analysis, ASL images were smoothed with a 3 ×3 gaussian kernel (*σ* = 2 voxels).

### 2.3 Voxel-wise analysis

In this section a compact notation is used for certain equation parameters which are also more commonly known under a different notation. To avoid any confusion we note here:

f = CBF (cerebral blood flow)
△ = ATT (arterial transit time)
*τ* = LD (labeling duration)
*w* = PLD (post-labeling delay)

The ASL signal was first separated into its blood, gray matter (GM) and CSF components by fitting the T_2_-decay to a tri-exponential function:

(1) $$S_{\text{total}\;}(TE)=S_{bl^{e}}-TE/T_{2bl}+S_{GM}e^{-TE/T_{2GM}}+S_{CSF}e^{-TE/T_{2CSF}}$$

**Fig. 2:**
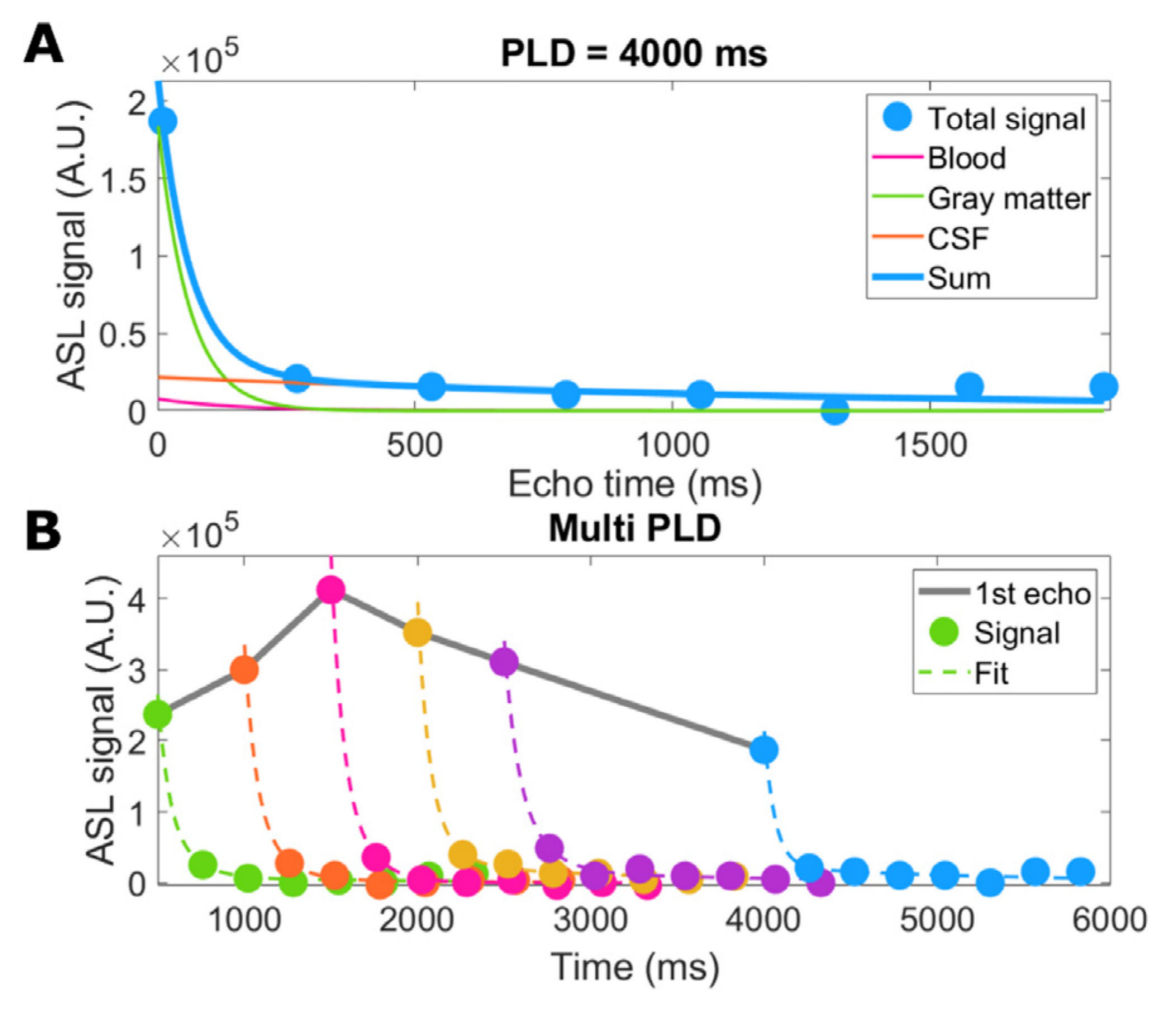
Example of single-voxel data fitted to the triexponential model. A shows the separation of the signal into three exponentials with T_2_ = 60 ms (GM), 150 ms (blood), and 1500 ms (CSF) for a single time point. B shows the fit to the sum of these three exponential components for all time points. Note that there are no error-bars as this dataset provided a single value for each voxel (any averaging over shots was done in image reconstruction).

With the fitted parameters S_bl_, S_GM_ and S_CSF_ being the signal at TE = 0 coming from the blood, GM and CSF compartments respectively, and T_2bl_, T_2GM_ and T_2CSF_ the T_2_ values associated with these compartments, which were taken from the literature (150 (Chen and Pike, 2009), 60 (Gelman et al., 1999), and 1500 (Spijkerman et al., 2018) ms for arterial blood, gray matter and CSF respectively). Note that the analysis was tailored to the blood, GM and CSF compartments and white matter signal was assumed to be negligible, since white matter ASL signal is expected to be very low for our protocol (due to the low CBF, long arrival times and short T_1_ preventing signal accumulation at longer labeling durations), in addition, T_2_ values of white and gray matter are similar and therefore difficult to distinguish with this approach (Wansapura et al., 1999). The fitting resulted in three maps containing the signal from each compartment, at every time point. This fitting procedure is shown in Fig. 2 as applied to signal from a single voxel situated in the cortex. In panel a, the separation of total signal into three exponential components is shown for a single time point, and in panel b the fit of the total signal is given for all time points.

This function results in an estimate of the compartmental origin of ASL signal through time, hindered by the length of the echo train (see Fig. 2b), during which exchange between compartments can occur. Additionally, it does not provide information about the dynamics of exchange between compartments. To achieve this, a compartmental dynamic model was introduced to fully describe the evolution of the signal through PLDs and TEs.

**Fig. 3:**
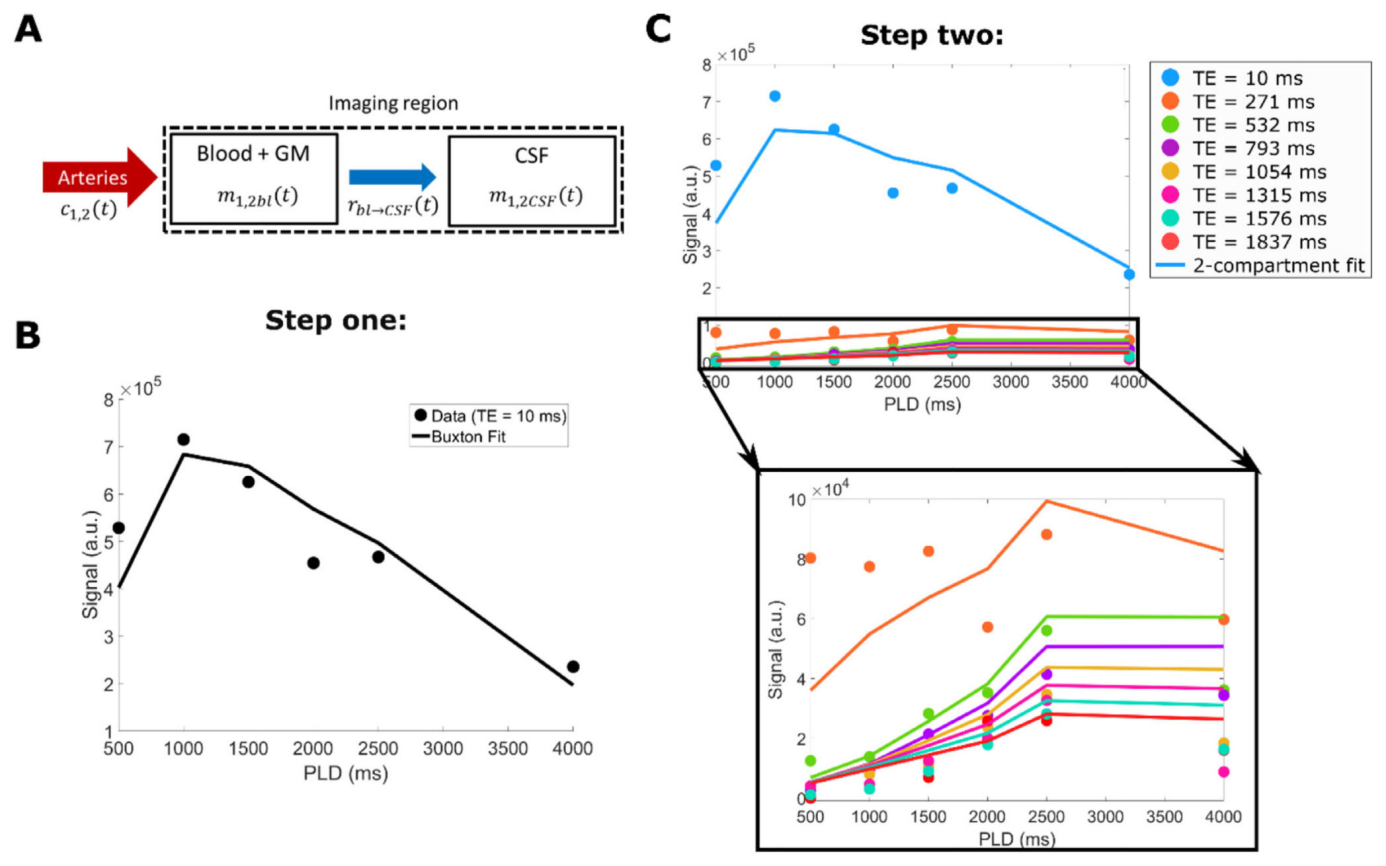
Explanation of the compartmental model fitting procedure. The schematic in A) represents the design and assumptions of the mathematical model, including the functions which describe the input (c(t)), exchange (r_bl->CSF_(t)) and decay (m(t)) of signal in and between compartments. B) shows the first fitting step: the data from the first echo time (TE = 10 ms) is fitted to a Buxton-type model to extract CBF and ATT. Then, as a final step (C) all 48 datapoints (for all PLD/TE combinations) are used as input in the two-compartment model to fit T_bl->CSF_ (with CBF and ATT fixed).

This model was inspired by the one proposed by Gregori et al. (Gregori et al., 2013), but modified to make it appropriate for PCASL rather than pulsed ASL. For this, we identify two compartments: the CSF, and a combination of blood and gray matter, which can also be described as the slow and fast decaying T_2_ compartments, with T_2_s of 1500 ms, and 100 ms (an average value for blood and gray matter), respectively. The blood and gray matter were combined because their short T_2_ values compared to the echo times used in this experiment made them difficult to separate. The model assumes instantaneous mixing within compartments (i.e. the exchange of water from blood to GM occurs on a much faster scale than blood to CSF and the blood/GM compartment can be considered homogenous), no outflow, unidirectional exchange from the blood to the CSF compartment, and instantaneous exchange of water between the blood and CSF (i.e. there is no delay between the arrival of water in blood and the beginning of exchange into the CSF, this is a common assumption in perfusion models which should hold true for blood-to-CSF water exchange which is mediated through the same aquaporin water channels as tissue perfusion). This is summarized schematically in Fig. 3a. In this section the subscript “bl” refers to the blood and GM compartment. The model is split into two parts, which represent the signal evolution before and after an excitation pulse. It consists of the convolution of an input function c(t), which corresponds to the arrival of signal in the voxel, with a residue function r(t) which represents the clearance of water from the compartment (in this case to cross into the CSF) and a magnetization relaxation function m(t), in which the signal decays with either the T_1_ or T_2_ of the compartment, before or after the excitation pulse, respectively. The integral form of the model before excitation is:

(2) $$S_{1bl}(t)=2M_{0}f{\int^{\text{​}}}_{0}^{t}c_{1}\left(t^{\prime }\right)r_{bl\to CSF}\left(t-t^{\prime }\right)m_{1bl}\left(t-t^{\prime }\right)\text{d}t^{\prime }$$

(3) $$S_{1CSF}(t)=2M_{0}f{\int^{\text{​}}}_{0}^{t}c_{1}\left(t^{\prime }\right)\left(1-r_{bl\to CSF}\left(t-t^{\prime }\right)\right)m_{1CSF}\left(t-t^{\prime }\right)\text{d}t^{\prime }$$

(4) $$c_{1}(t)=\{\begin{array}{l} 0,t<\Delta \\ \alpha e^{-\Delta /T_{1bl}},\Delta \leq t<\tau +\Delta \\ 0,\tau +\Delta \leq t \end{array}$$

(5) $$r_{bl}\to CSF(t)=e^{-t/T_{bl}\to CSF}$$

(6) $$m_{1(bl,CSF)}(t)=e^{-t/T_{1(bl,CSF)}}$$

Here M_0_ represents the equilibrium magnetization of blood, *α* is the labeling efficiency, T_bl->CSF_ is a time constant that describes the exchange of labeled water between the blood and CSF compartment, and T_1(bl,CSF)_ are the longitudinal relaxation times associated with the two compartments, set at 1650 and 4300 ms respectively. We use the (bl,CSF) notation for equations that have the same form for both compartments, to make the formulation more compact. In this part of the model, the time t is equal to the total time from beginning of labeling to excitation, i.e. *t* = *τ* + w.

After excitation, the equations are:

(7) $$S_{2bl}(TE)=2M_{0}f{\int^{\text{​}}}_{0}^{TE}c_{2}\left(t^{\prime }\right)r_{bl\to CSF}\left(TE-t^{\prime }\right)m_{2bl}\left(TE-t^{\prime }\right)\text{d}t^{\prime }$$

(8) $$S_{2CSF}(TE)=2M_{0}f{\int^{\text{​}}}_{0}^{TE}c_{2}\left(t^{\prime }\right)\left(1-r_{bl\to cSF}\left(TE-t^{\prime }\right)\right)m_{2CSF}\left(TE-t^{\prime }\right)\text{d}t^{\prime }+S_{1CSF}(\tau +w)m_{2CSF}(TE)$$

(9) $$c_{2}(TE)=\{\begin{array}{lll} 0, & TE<\max(\Delta -\tau -w,0) & \\ \alpha e^{-\Delta /T_{1bl}}e^{-TE/T_{2b}}+\frac{\delta_{\text{Dirac }}(0-TE)S_{1bl}(\tau +\omega )}{2M_{0}f} & \max(\Delta -\tau -w,0)\leq TE<\Delta -w & \\ 0, & \Delta -w\leq TE & \end{array}$$

$$m_{2(bl,CSF)}(t)=e^{-t/T_{2(bl,CSF)}}$$

With TE being the echo time (i.e. *t = τ* + *w* + TE) and *δ*_Dirac_ denotes the Dirac delta function. Note that the signal that is measured equals S_total_(t,TE) = S_2bl_(TE) + S_2CSF_(TE). For a complete solution to these equations, refer to the appendix. With the T_1_ as well as the T_2_ values fixed, there remain 3 parameters which can be fitted in this model: CBF, ATT, and the exchange time from the blood/GM to the CSF T_bl->CSF_. By inserting data from all time points and all TEs (6 LD/PLD combinations x 8 TEs =48 data points per voxel), maps of the three fitted parameters can be generated. From our model and these maps, the signal coming from each compartment could be recalculated at arbitrary time points (any LD and PLD combination) to create videos which dynamically show the signal evolution and distribution in the brain in a more intuitive manner (combined changes in LD and PLD used in this study make interpretation difficult).

In order to stabilize the fit of this complex model, it was performed in two steps: first, the data from the first TE image and all LD/PLD combinations was fitted to a conventional Buxton model (Buxton et al., 1998) to extract the CBF and ATT (Fig. 3b). Then, these values were fixed in the dynamic compartmental model, which was used only to fit the third parameter, T_bl->CSF_, by including all 48 data points of the ASL signal (Fig. 3c). The M_0_ value was obtained from the M_0_ scan by averaging the signal over the gray matter mask and dividing by the blood-brain partition coefficient, *λ =* 0.98 ml/g (Herscovitch and Raichle, 1985) (i.e. a single average value was used for all voxels).

Three ROIs were created to compare calculated parameters in the choroid plexus and the CSF space surrounding the cortex (subarachnoid space), as well as the white matter as a control region where no CSF exchange is expected. For this, the choroid plexus was manually delineated on the CSF mask by isolating the posterior part of the lateral ventricles and the subarachnoid space ROI was defined by removing the ventricles from the CSF mask. The white matter mask was obtained by removing the gray matter and CSF from a brain mask.

The data and code used in this project are confidential but may be provided upon request when signing a data use agreement.

## Results

### 3.1 ASL signal and validation

**Fig. 4:**
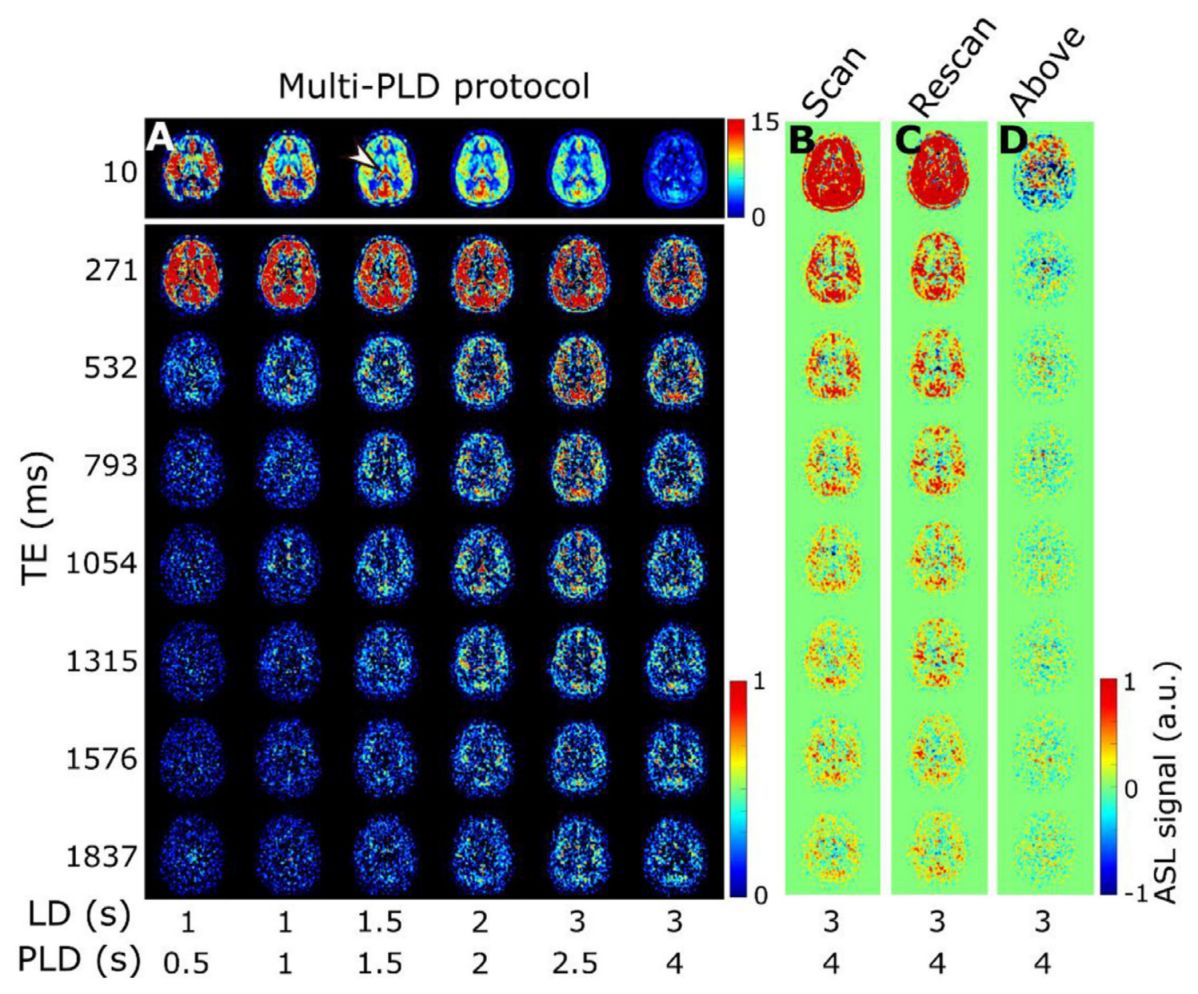
ASL signal and validation. A) ASL signal from the multi-PLD protocol for subject 1 in a single slice at all inflow time points (left to right) and echo times (top to bottom). The first echo is shown in a different scale for better visualization. In B the last column of A is repeated with the inclusion of negative signal for comparison to the validation scans. The arrowhead points to the choroid plexus (present in all images but especially visible at this time point in the perfusion image). C shows the ASL signal of the reproducibility scan (note that images are not co-registered and therefore slices may not correspond perfectly) and D shows the signal with the labeling plane above the brain.

**Fig. 5:**
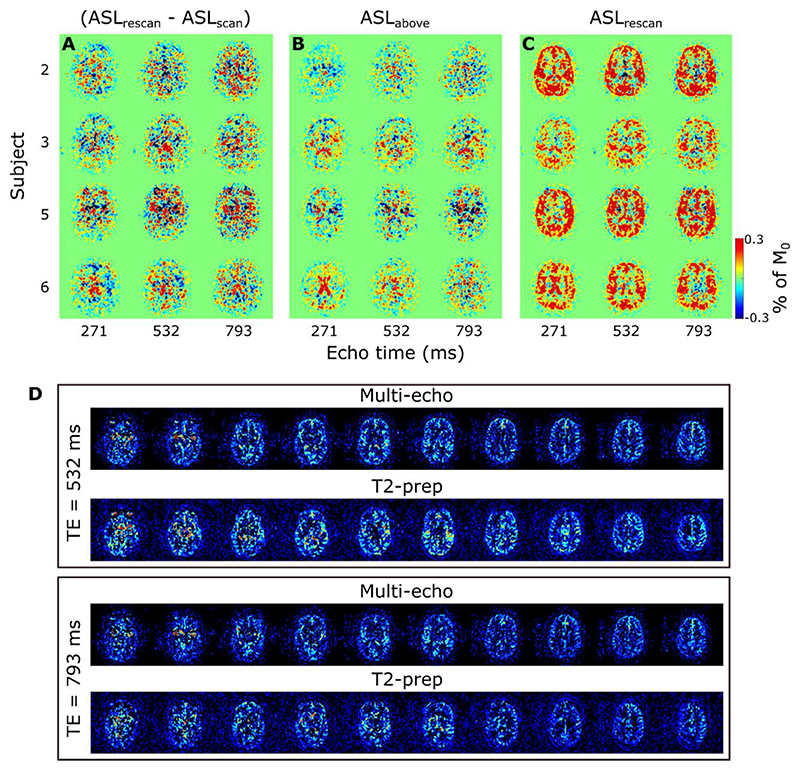
ASL signal differences between the reproducibility scans (a), the scan labeled above the brain (b), and the actual measured signal (c), in a single slice at three TEs for all four subjects who underwent the validation scan session. Values are shown as a percentage of the average M_0_ (calculated separately for each echo). D shows the results of the T_2_-prep experiment, for LD/PLD = 3/2.5 s, comparing the signal in ten central slices at TE = 532 ms and TE = 793 ms for the multi-echo readout, vs the single-echo readout with T_2_-prep adjusted to reproduce the same echo times.

ASL images for one subject, in a single, transverse slice intersecting with the choroid plexus in the lateral ventricles, are shown in Fig. 4. In 4a, the results of the multi-PLD experiments are displayed for all time points and all echo times. The first echo time is consistent with more typical ASL measurements: the signal is seen transitioning from the large arteries in the early time points to then perfusing into the tissue and finally decaying at long PLDs. Looking at the longer echo times (≥ 793 ms) informs us on the presence of signal in the CSF, as only a very long T_2_ allows signal to persist at those TEs. Starting at PLD =1.5 s, this long-T_2_ signal begins to appear, increasing and peaking around PLD =2.5 s. Interestingly, this signal is not confined to the choroid plexus (the structure is highlighted by an arrowhead in the image where it is most visible in the top row of Fig 4a), nor does it appear to originate from there, as might be expected based on the current consensus hypothesis of CSF circulation (Milhorat, 1975); in fact, the CSF-ASL signal is fairly well distributed around the cortex. Similar results were seen in 6 subjects with varying age and sex (see supplementary figure S1).Fig. 4b-d compares the long PLD results of the initial multi-PLD protocol (b is the same as the last column of a) to the reproducibility scan (c) and the scan with the labeling plane above the head. Even though there is not a perfect match of the slices in b and c, the intensity and pattern of the signal are qualitatively comparable. For a more quantitative comparison, Fig. 5 shows the difference between the ASL signal for the original scan from the multi-PLD protocol vs the repeated scan (reproducibility experiment, in a), and contrasts this to the ASL signal obtained when labeling above the head (b) and the repeat scan (c). The relative difference between signals in the reproducibility experiment (a) is shown to be small, with no distinct spatial pattern, and no bias towards positive or negative values, meaning that differences can largely be attributed to noise and errors in registration. For the scan labeled above the brain, we observe a similar amount of signal as the test-retest difference, also with little spatial coherence, i.e. the amount of signal is on the same order of magnitude or lower than the natural fluctuations that occur between repeated measurements. For comparison, the actual ASL signal is shown in c, to highlight the difference in scale between real signal and noise. Finally, the results of our stimulated echoes experiment are shown in d. The two TEs employed are long enough to isolate CSF signal, and we can observe similar signal intensity (average signal ± SD (A.U.) in the extraventricular CSF for TE = 532 ms: 2.2 ± 1.9 and 2.1 ± 2.0 for the multi-echo and T_2_-prep sequences respectively, and for TE = 793 ms: 1.8 ± 1.6 and 1.1 ± 1.8) for each echo with the multi-echo and the T_2_-prep methods. Since background suppression had to be modified in the T_2_-prep sequence to optimize static tissue nulling at the beginning of the module instead of the beginning of readout, the apparent reduction in SNR for the T_2_-prep method is expected. We also performed a simulation of the effect of the imperfect, 160° refocusing pulses on the signal evolution through the echo train (shown in supplementary figure S2) for blood, gray matter and CSF using the extended phase graph formalism (Weigel, 2015) and found it to be minimal in comparison with expected signal fluctuations due to noise. From this, we conclude that the CSF-ASL signal that we observe could only originate from water that is labeled in the brain-feeding arteries and transported from the blood to the CSF.

### 3.2 Triexponential fit

**Fig. 6:**
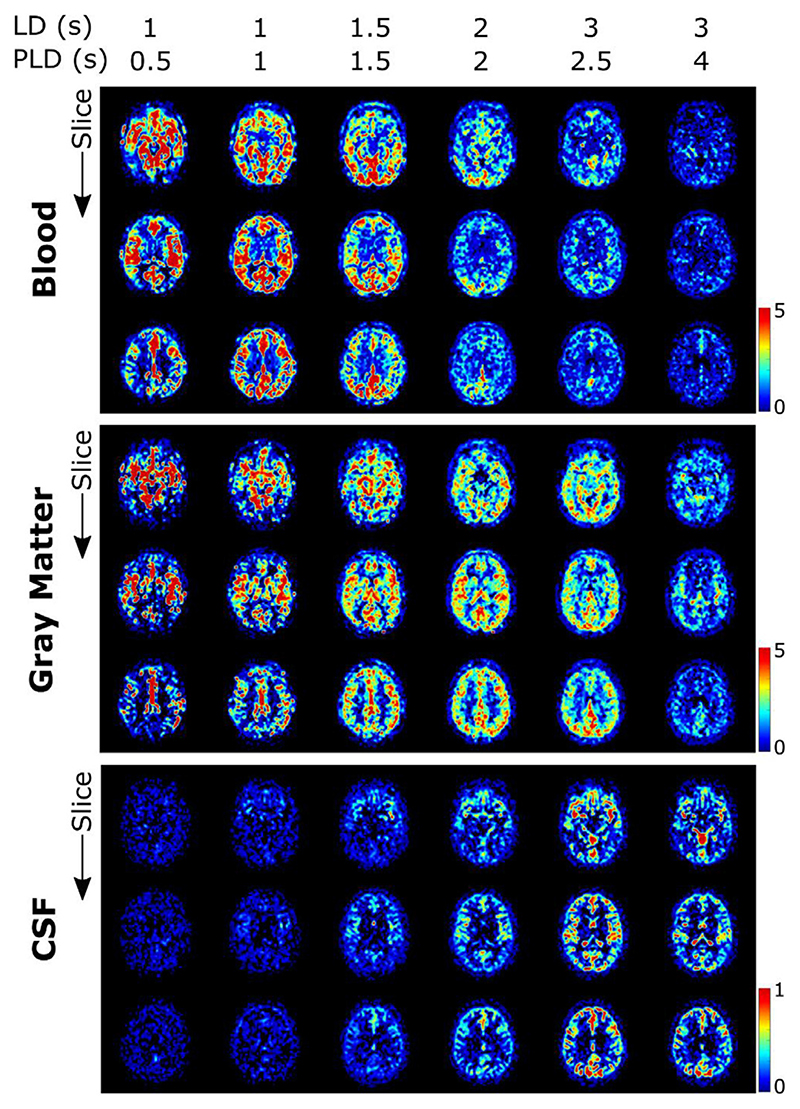
Results of triexponential fit in subject 1. The signal originating from each of the three compartments is shown separately (note that because the CSF signal is much lower, a different scale is used). Maps are given for three slices, one intersecting the circle of Willis (top), one intersecting the choroid plexus (middle) and one higher in the brain (bottom). Different time points are shown with increasing LD/PLD from left to right.

The results of the triexponential fit of the ASL signal for one subject are presented in Fig. 6. These maps show the total amount of signal that originates from each compartment according to their respective T_2_ and are equivalent to the zero crossing of the three exponentials of Fig. 2a. Some overlap can be seen between the blood and gray matter maps. This is explained by the close values of the T_2_‘s of GM and blood relative to the employed echo times of our sequence. Most relevant for this study however is the separation of the CSF signal from the other two compartments, which appears to be achieved well, with a very distinct pattern both spatially and temporally, corresponding to the ventricles and the subarachnoid space around the cortex. The amount of overlap between the blood and gray matter signal due to their relatively similar T_2_ values (compared to CSF) informed our decision to combine them into a single compartment for the dynamic model, using an intermediate T_2_ value.

### 3.3 Dynamic compartmental model

**Fig. 7:**
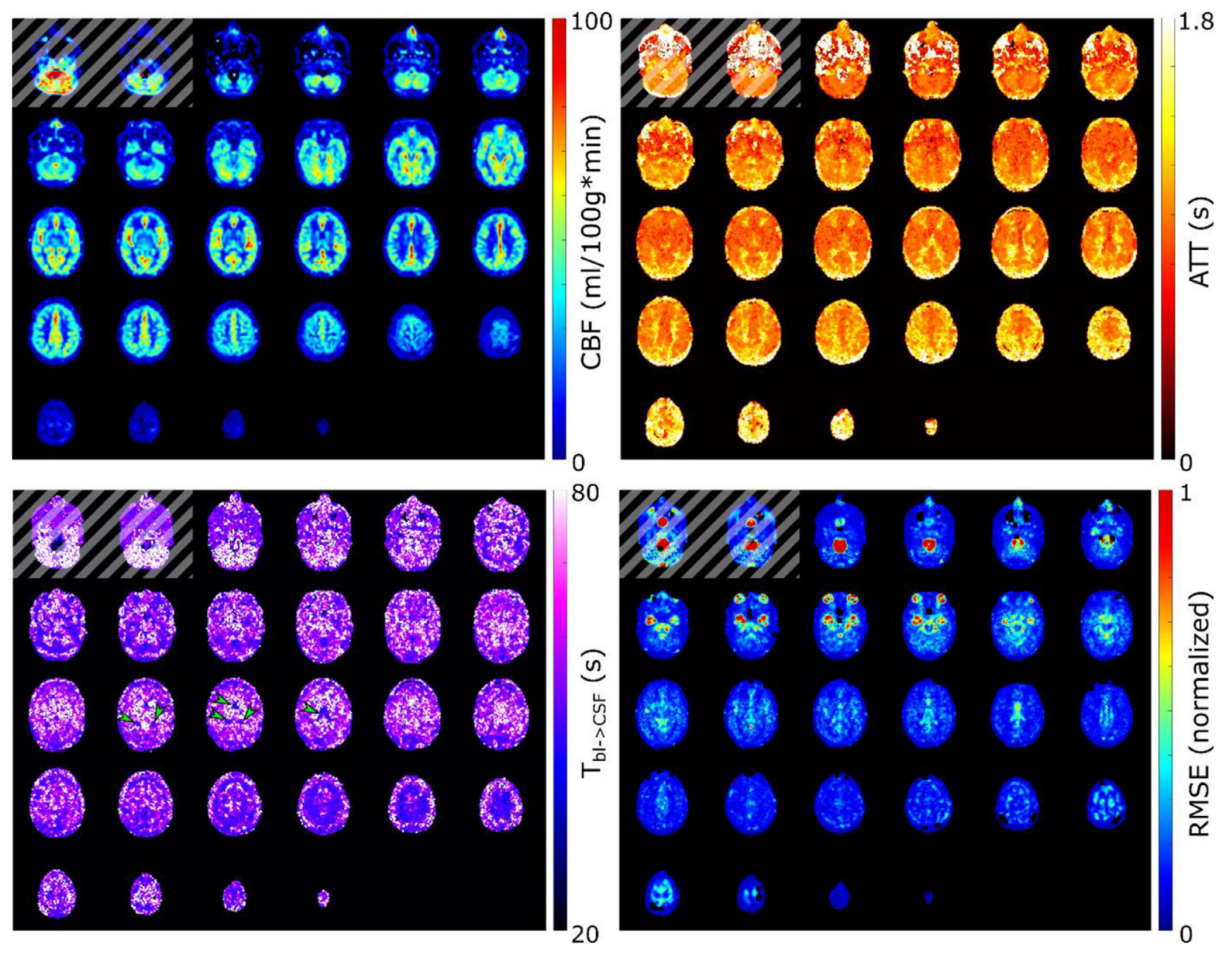
Whole brain maps of CBF, ATT, T_bl->Csf_, and RMSE (root-mean-square error of the residuals for echoes 3–8) for subject 1 obtained using the dynamic compartmental model fit. Note that the ASL labeling plane intersects with the bottom of the imaging volume, resulting in erroneous values in the lower slice(s) (shown with a striped overlay). The RMSE is normalized to the same scale as the long-echo ASL signal of Fig. 3a. Arrowheads point to the choroid plexus in the T_bl->CSF_ map.

The dynamic compartmental model (outlined in Fig. 3) provides a more complete picture of the dynamics of exchange at play between the compartments. Three parameters are fitted: CBF, ATT, and T_bl-> CSF_, shown in Fig. 7, where whole brain maps are given for one subject. CBF and ATT are commonly quantified in ASL studies, and the patterns and values observed here are consistent with literature (Chen et al., 2012; Alsop et al., 2015), with high CBF in the cortical gray matter and low CBF in white matter areas. ATT is short around the large arteries (circle of Willis, penetrating arteries, anterior cerebral artery), longer in the posterior flow territory compared to the anterior, and longest in the white matter. The T_bl->CSF_ image is noisier but it is possible to distinguish areas containing CSF (choroid plexus (arrowheads), around the cortex, and in higher slices) by their shorter values.Fig. 7 also shows the root-mean-square error (RMSE) of the fit calculated on echoes 3–8 (as an indication of the fit of the CSF signal), normalized to the same scale as the long-echo ASL signal of Fig. 4a. The RMSE is highest in areas with large vessels and the eyes, as expected, with lower values in gray matter and CSF regions.

**Fig. 8:**
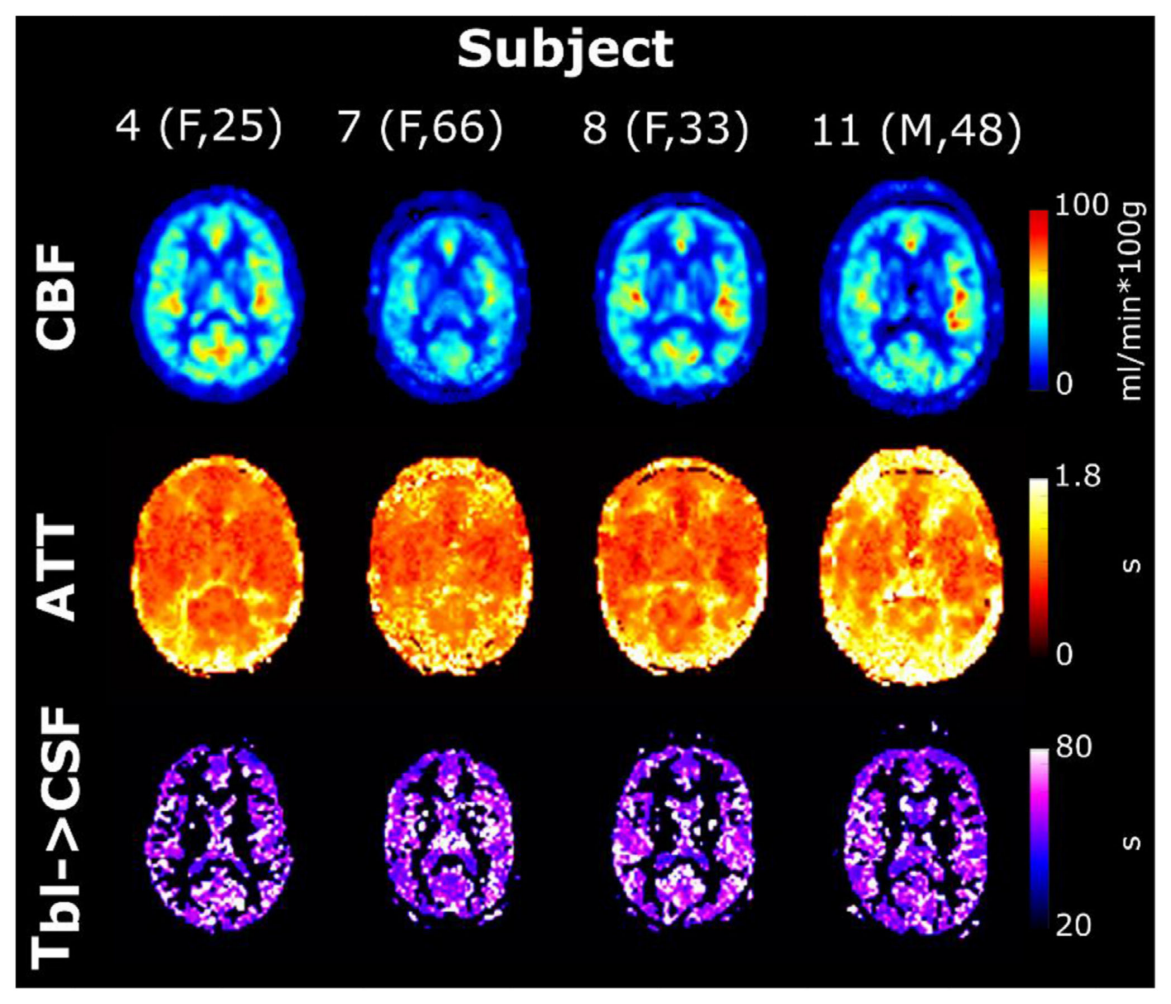
Single-slice parameter maps for four subjects with variable age and sex. T_bl-> CSF_ is shown only in the gray matter and CSF masks for easier visualization (white matter areas are particularly noisy).

**Fig. 9:**
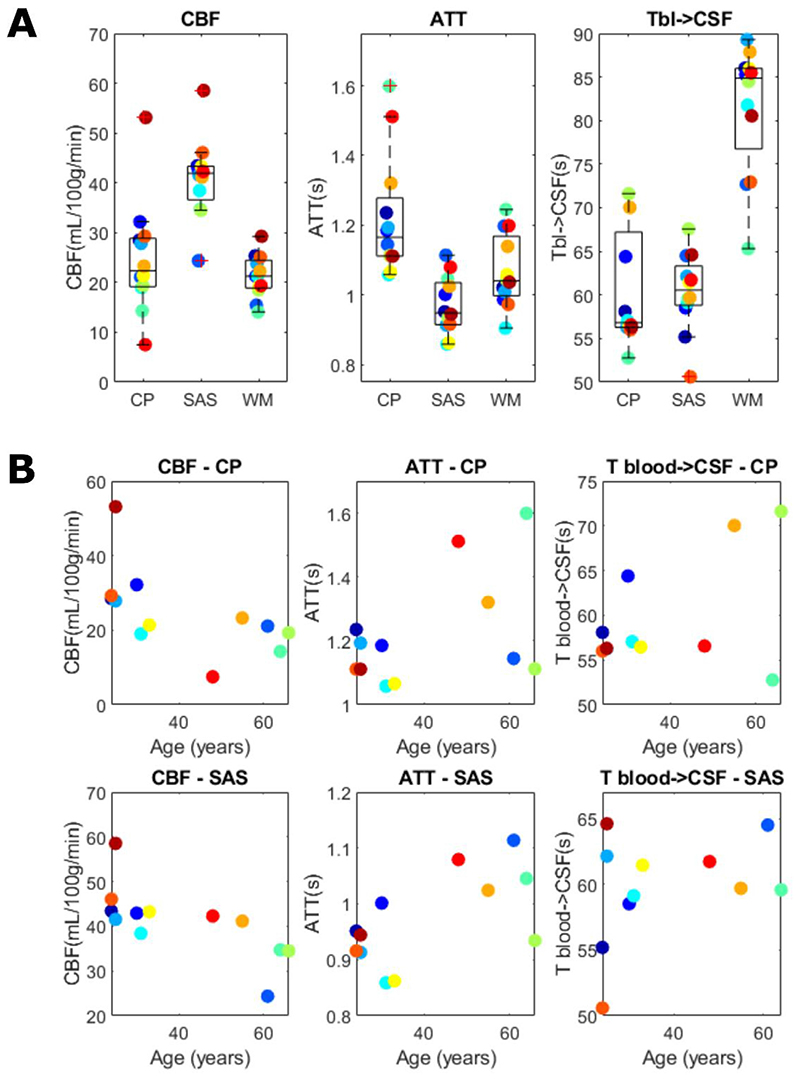
Dynamic compartmental model parameter averages per subject for (a) the choroid plexus (CP), subarachnoid space (SAS) and white matter (WM) ROIs and (b) plotted as a function of age for the CP and SAS ROIs.

In Fig. 8, the same parameter maps are shown for four subjects. Notice the decreased CBF and longer ATTs present in the older subjects (7 and 11) consistent with studies done in aging brains (Alsop et al., 2015; Clement et al., 2018). Here the T_bl->CSF_ maps are masked (including the gray matter and CSF masks) to better appreciate the values without the noise originating from the white matter region.Table 1 summarizes the average values of the fitted parameters for all subjects in the choroid plexus, subarachnoid space, and white matter ROIs in a single slice to avoid bias along the slice direction (for subject 1 shown in Fig. 7, slice 15 from the bottom was chosen, and this location was replicated as closely as possible in other subjects). Values for CBF and ATT in the choroid plexus and white matter are consistent with literature (Zhao et al., 2020) while values in the cortical CSF are more typical for gray matter, likely due to partial volume effects and the proximity of penetrating arteries. These same results are shown graphically in Fig. 9. In a, boxplots are shown with the average values for all subjects for all three ROIs. No statistical testing was performed because of the small sample size, however this highlights the differences in the fitted parameters for these ROIs. We note the elevated T_bl->CSF_ in the white matter ROI, consistent with a lower amount of blood to CSF exchange, while these values are very similar in the choroid plexus and subarachnoid space. The WM ROI is included as a control region where we do not expect to observe blood to CSF exchange to compare with the values of other ROIs (since there are no existing literature values for this parameter). This is the case both because there is little to no overlap of white matter and CSF, and because the ASL signal in white matter is very low (leading to the large standard deviations seen in table 1). Values were also plotted in b (for only the two ROIs where CSF exchange is expected) as a function of age, and while again we do not have sufficient power to establish a statistically significant trend, from visual inspection of these data we do notice an apparent increase in T_bl->CSF_ with age, while CBF and ATT appear to decrease and increase with age respectively, as expected.

**Fig. 10:**
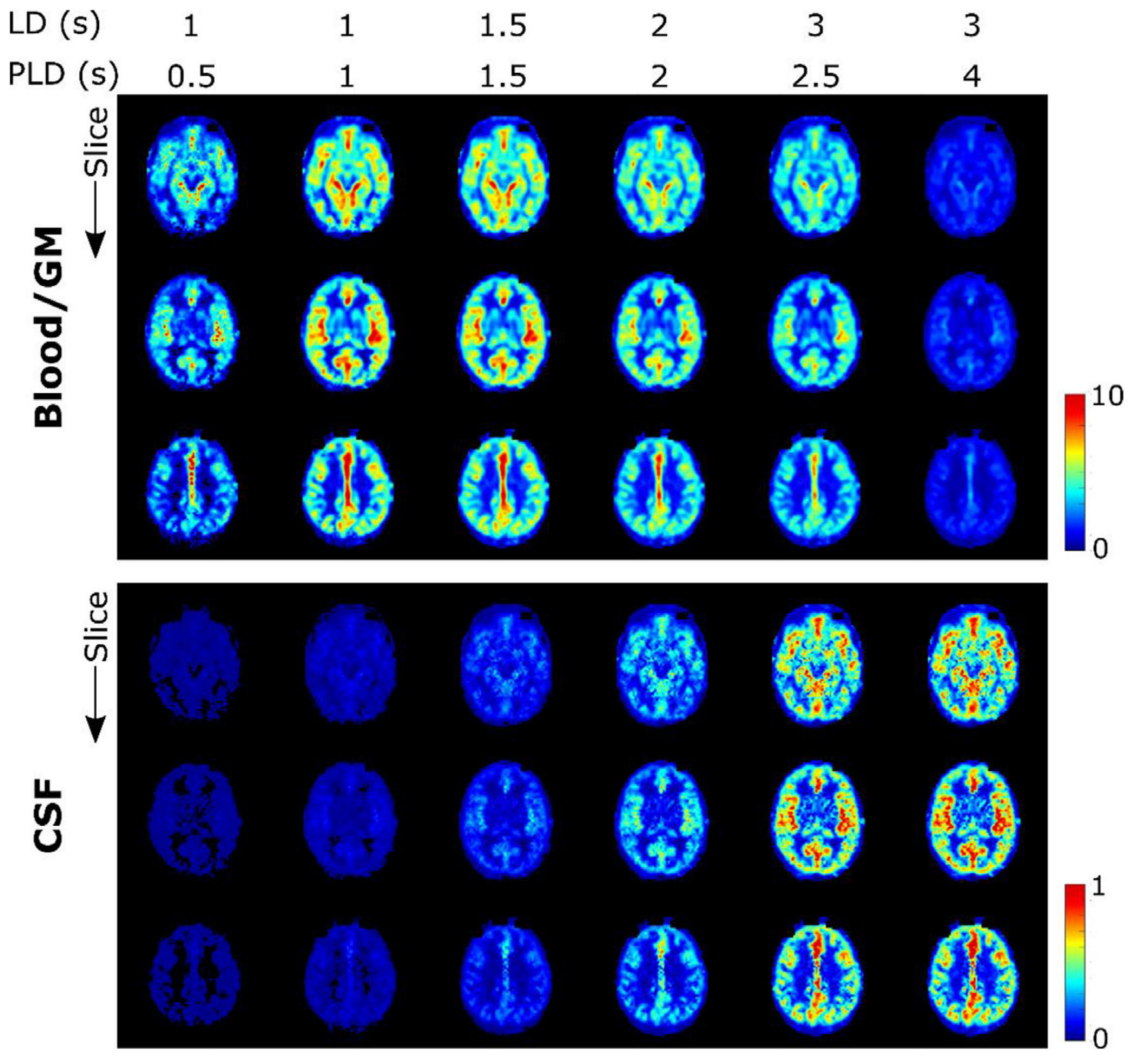
Reconstructed blood (+GM) and CSF fractions from the fitted parameters of Fig. 7, simulating the signal at all experiment time points for an artificial echo time of 0 ms. Note the different scaling of the CSF fraction.

Fig. 10 shows the compartmental signal fraction maps artificially reconstructed from the parameter maps of Fig. 7 at the LD/PLD combinations used in this study and a fictitious echo-time of TE = 0 ms. This corresponds to a similar result as that given in Fig. 6, this time using the dynamic compartmental model as opposed to the triexponential model, with the blood and gray matter signals combined (summed) into one. Upon visual inspection, the results of these two techniques are fairly similar. The location and amount of blood/GM signal in Fig. 10 is consistent with the sum of the two compartments in Fig. 6 (note the different scales used), as well as with the existing ASL consensus predicting the peak of GM perfusion to appear at a PLD of around 1.8 s (Alsop et al., 2015)). High spatial correspondence between the CBF map and the presence of gray matter is also consistent. The features of the CSF-signal map are in general comparable, but some important differences can be observed. While the timings of arrival of signal in this compartment are preserved, the signal is more evenly distributed across the cortex in Fig. 10. A notable difference between the two techniques is the presence of CSF signal in the frontal horn of the ventricles (Fig. 6, middle slice), which is absent in the case of the two-compartment dynamic model.

The same model was used to reconstruct the signal for LD = 3 s and with a PLD increasing from 0 to 5 s (again a TE of 0 ms was used) based on the maps of the fitted parameters (see supplemental video figure S3). This simulates dynamically the passage of a 3 s bolus of labeled water through the compartments, where we can see the early peak and decay of signal in the blood/gray matter, and the late arrival and plateau of water signal in the CSF. Although these simulations may not be a perfect representation of reality, they show the far-reaching distribution of blood-CSF exchange sites which are present throughout the cortex, and give a helpful visualization of the transport of water in space and time.

## 4 Discussion

In this study, we used a modified multi-PLD ASL protocol to measure the exchange of water from the blood to the CSF. Our main conclusions were as follows: firstly, imaging CSF-ASL signal in the human brain is feasible and shows sites of exchange that extend beyond the choroid plexus; secondly, our results indicate that CSF signal can be isolated from other compartments based on its distinctly long T_2_ and that a simple dynamic compartment model can provide a reasonable fit to the observed signal evolutions.

The presence of CSF-ASL signal is shown in Figs. 4 and 5 (and in supplementary figure S1 for more subjects). The presence of sustained signal in the long PLD scans at echo times ≥793 ms, considering the T_2_s of arterial blood (150–250 ms (Chen and Pike, 2009)), gray matter (60–100 ms (Stanisz et al., 2005)) and CSF (1500–1800 ms (Spijkerman et al., 2018)), can only mean that this signal is originating from the CSF. This signal is also evolving through time i.e. it increases and plateaus for longer PLDs, which is a behavior that is unlikely to happen if this signal were artefactual (in which case it would appear with no relationship to labeling parameters). The analysis shown in Fig. 5 further confirms this. Differences in ASL signal between identical scans acquired on different days show that their results are largely similar (the difference amounting to (physiological) noise), and comparing this to the scan labeled above the head shows that essentially no CSF-ASL signal is created unless it originates from the arterial labeling. Most interestingly, whereas we expected the CSF-ASL signal to be concentrated in the choroid plexus area, especially in light of the previous study performed in mice (Evans et al., 2020), here it was found to be well distributed around cortical areas as well. The CSF signal fraction maps calculated from the triexponential method (Fig. 6) and the dynamic compartmental model (Fig. 10) bring further confirmation of CSF-ASL signal around the cortex. This therefore seems to indicate the presence of blood-CSF water exchange sites around the cortex, in the subarachnoid space. This observation could be attributed to the existence of aquaporin-1 water channels (found in high concentrations in the choroid plexus and responsible for CSF secretion (Day et al., 2014)) in the leptomeningeal (i.e. pial) vasculature, which has been recently shown in rodents (Li et al., 2020). These vessels are situated in the subarachnoid space which is filled with CSF and wraps around and into the fissures and sulci of the cortex, which would explain the co-localization of the signal in those areas. Although previous experiments in mice (Evans et al., 2020) did not observe CSF-ASL signal outside of the choroid plexus (see Peer Review file in Supplementary Information in (Evans et al., 2020)), this discrepancy could be due to the low subarachnoid-CSF volume in mice combined with limited image resolution, or differences in the polarization of aquaporin water channels between humans and mice, which has previously been shown for AQP-4 (Eidsvaag et al., 2017).

Our second conclusion is that CSF signal can be isolated from other compartments based on T_2_ and our proposed dynamic compartmental model adequately describes the signal evolution and therefore exchange between compartments. The triexponential fit (Fig. 6) shows overlap between the blood and GM signals, but not CSF. This is because the very long T_2_ of CSF results in a distinctive signal evolution through the echo train (which was almost 2 s in length), with TEs chosen specifically to be tailored to this compartment. The dynamic compartment analysis uses more parameters to fully describe the exchange between compartments, but the separation of signal from these compartments is fundamentally dependent on the assumed T_2_s. Including the compartmental T_2_ values as unknown fitted parameters resulted in unstable fits. Notably, the triexponential fit (Fig. 6) shows high CSF signal in the frontal horn of the lateral ventricles (middle slice) while the dynamic analysis (Fig. 10) does not. Since this area does not contain a choroid plexus, this may point to the limits of the simple triexponential function to properly differentiate between areas of exchange as opposed to areas simply containing CSF. It should also be noted that the signal fractions in Fig. 10 are calculated from the parameter maps of Fig. 7. This additional step introduces more uncertainty as the errors in parameter estimation are carried over to the signal maps. These maps and the supplementary video S3 are helpful visualizations, however they may not perfectly reflect accurate exchange dynamics.

While there is no existing measurement of T_bl- > CSF_ to compare ours to, the contrast exhibited between the ventricular CSF containing choroid plexus tissue/subarachnoid areas and the white mat- ter/ventricular CSF which does not contain choroid plexus tissue in Figs. 7 and 8 shows a distinction between areas with and without blood- to-CSF exchange (longer values indicate little to no exchange). The parameter values plotted in Fig. 9 further confirm these differences. The RMSE of the fit (for echoes 3–8) was also computed, and showed relatively uniform error except in voxels containing large vessels (circle of Willis and middle cerebral arteries for example), which is expected as the model is not meant to describe bulk flow of blood. We also observe a higher RMSE in ventricle regions directly adjacent to the choroid plexus, which implies that different mechanisms are at play, such as mixing of labeled blood water that has been delivered to the CSF in the choroid plexus with neighbouring voxels. This effect is expected to be largest in the ventricles where CSF mobility is high.

There are a number of limitations with the imaging and analysis methods used in this study. First, we are limited in the number of time points (LD/PLD pairs) that can be acquired within one scan session, especially later time points which require increasingly long sequences to perform. Here we wanted to sample the entire passage of the bolus from its arrival in the voxel to its transition into CSF. This meant that a compromise had to be achieved, and only the initial phase of water influx into the CSF compartment could be sampled. In this proof of concept study, it was important to measure the passage of the labeled bolus over long labeling duration/PLD times in order to distinguish CSF from blood signal with greater certainty. Now that the signal from labeled blood water that has been delivered to the CSF has been shown to exist and be readily measurable, and preliminary measurements of T_bl->CSF_ have been performed, future protocols could adapt LDs and PLDs to be more specific to the CSF compartment and thereby minimize total scan-time. In addition, time-encoded ASL sequences (Wells et al., 2010; Teeuwisse et al., 2014) could be devised to acquire these images in a more efficient manner, although durations of sub-boli should remain long enough to guarantee sufficient SNR. The dynamic two- compartment model showed reasonable agreement with the data, however we could not escape the pitfalls of fitting such complex models, and we opted for a two-step fitting process with the simpler Buxton model as the first step. We note that the estimates of T_bl->CSF_ are long (> 50 s) in comparison to labeling parameters (LD + PLD <10 s) and therefore should be interpreted as an order of magnitude rather than a precise measurement. Other model assumptions could be affecting the value of this parameter, such as the assumption that all labelled water eventually is transported into the CSF (this artificially increases T_bl->CSF_ which is likely to be shorter when employing a more complex model with additional compartments), whereas other considerations such as bulk flow of blood and mixing of CSF could provide a more complete, but also more complex, picture. Because the T_2_s of blood and gray matter are so short and close to each other in comparison to the TEs of the imaging sequence, we have opted for a two-compartment model, which combines the blood and gray matter into one compartment. A three-compartment model, which includes a T_bl->GM_ parameter, could be fitted, provided that a range of shorter TEs be also acquired with the protocol, for example by combining our approach with a T_2_-prep module. (Schmid et al., 2015) Moreover, our compartmental model (as well as the Buxton model used to fit CBF and ATT) does not include macrovascular signal, therefore the modelled parameters show incorrect values (higher CBF, lower ATT, longer T_bl->CSF_) around the large arteries (circle of Willis, anterior cerebral artery, etc.), as shown in Fig. 7d, where the RMSE was highest in those areas. This is further complicated by the fact that, with our multi-echo acquisition design, long echo times include higher levels of vascular crushing from imaging gradients. A sequence design including explicit flow crushing gradients could alleviate these issues. Additionally, some erroneous signal was observed as fold-over artifacts from the eye signal (especially in longer TEs), and a negative signal in the ventricles, outside of the choroid plexus, which was seen inconsistently in some of the subjects but did not seem to affect our analysis. We also note that at very long TEs the signal intensity may be comparable to the noise floor of the acquisition leading to a possible overestimation or instability of this signal. Although we employ spatial smoothing to reduce noise, the additive Rician noise found in magnitude images is amplified by the ASL subtraction procedure, introducing bias in the CSF signal. However, our results when measuring ASL signal labelled above the brain (Fig. 5B) also show that signal still dominates the noise at these longer echo times. Future studies may include more sophisticated noise filtering techniques such as those used in diffusion imaging (Zhang et al., 2021;Andersson, 2008). The spatial smoothing applied in this work, while removing some noise from our data, also has the added caveat of increasing partial volume issues, both in the choroid plexus and the subarachnoid CSF, as both of these structures are small/thin compared to our imaging resolution (3 × 3 mm in-plane) and smoothing kernel (*σ*= 6 mm). Another source of error for our model could come from the assumption that there is no spatial mixing of labeled water in the CSF between voxels. In reality, CSF flow is pulsatile and can reach velocities up to 8 cm/s in areas such as the cerebral aqueduct (Battal et al., 2011). However, this applies mostly to areas containing large CSF pools (ventricles, aqueduct), while CSF flow is slower in other areas such as the perivascular spaces (experiments done at our facility, for example, use bipolar gradients with crushing velocity of 0.5 cm/s to measure CSF mobility in perivascular spaces). This could still influence the signal at our temporal and spatial resolution, in particular around the choroid plexus in the ventricles, where we also did observe higher RMSE, and this could be taken into account in the future by including a spatial mixing term in our model. Finally, upon further investigation of the dynamic compartmental model fitting results, it appears that the signal in the last time point at longer echo times is consistently overestimated by the model. As can be seen in Fig. 3c (in the insert), the signal tends to decrease at that time point rather than remaining constant as the model predicts. As this was a common observation, it could indicate the presence of outflow from the CSF compartment, for example if the blood-CSF exchange is bidirectional instead of unidirectional. This exchange from the CSF back to the blood could be added to our model in the future, at the cost of increased complexity.

This proof of concept study leaves several questions to be answered. For one, it is not possible from our results to determine whether there is net inflow of water from the blood to the CSF, i.e. what we could characterize as CSF formation. As discussed above, there is some indication of outflow from the CSF compartment which could be attributed to exchange back into the blood. Further studies with a better sampling of the CSF signal in time are needed to confirm this. Also, we currently speculate that the observed ASL-CSF signal in the subarachnoid space is coming from the leptomeningeal and pial vasculature because of the findings of a recent study (Li et al., 2020) observing exchange sites for CSF in those vessels. In order to confirm this, super-selective ASL could be leveraged to label individual vessels judiciously as they branch out into this area of the brain. In addition, the results of this study have potential implications for the application of partial volume correction in ASL, which typically assumes negligible signal in the CSF compartment, which we have shown here to be incorrect. It remains to be seen how large the effect of including CSF signal in partial volume corrections would be, however it is worth noting that the CSF signal at the longest PLD appears to reach about 20% of the perfusion signal, which also has implications for the traditional interpretation of ASL-CBF uniquely as the rate of delivery of blood to the capillaries and tissue. Finally, the CSF signal could be isolated by using a single long (> 700 ms) echo time instead of 8, which could shorten the length of the readout and therefore the TR, however this would prevent the full description of dynamics of exchange between compartments.

In conclusion, we have shown that imaging of CSF-ASL signal is feasible in the human brain, and that isolating this signal and describing its exchange with surrounding compartments can be achieved with the appropriate imaging parameters (LD/PLD and TE). We found that ASL signal is present in both ventricular CSF proximal to the choroid plexus and the subarachnoid space, it is approximately 5 times lower than the traditional perfusion signal measured in the gray matter, and we estimate a blood-CSF water exchange time (T_bl->CSF_) in the range 50–70 s. This work supports the emerging view that the “third circulation” theory, in which CSF is secreted exclusively at the choroid plexus and later reabsorbed in arachnoid granulations, is an insufficient explanation for CSF physiology and brain clearance. Indeed, we add to a mounting body of evidence that CSF exchange occurs outside of these key areas and is widespread throughout the brain.

## Supplementary Material

Supplementary material associated with this article can be found, in the online version, at doi: 10.1016/j.neuroimage.2021.118755.

Supplementary Material

Supplementary Movie

Appendix

## Figures and Tables

**Table 1:** Summary of parameter values in the choroid plexus (CP), subarachnoid space (SAS) and white matter (WM) ROIs for all subjects in a single slice intersecting the choroid plexus. All values are given as mean ± SD.

Subject # (sex, age)	CBF (ml/100 g*min)			ATT (s)			T_bl->CSF_ (s)		
	CP	SAS	WM	CP	SAS	WM	CP	SAS	WM
1 (F, 24)	28 ± 13	43 ± 17	25 ± 11	1.2 ± 0.3	1.0 ± 0.1	1.0 ± 0.2	58 ± 17	55 ± 11	86 ± 84
2 (F, 30)	32 ± 13	43 ± 15	21 ± 8	1.2 ± 0.3	1.0 ± 0.2	1.0 ± 0.2	64 ± 23	59 ± 13	85 ± 82
3 (F,61)	21 ± 6	24 ± 10	15 ± 5	1.1 ± 0.2	1.1 ± 0.2	1.2 ± 0.2	75 ± 34	64 ± 34	73 ± 42
4 (F, 25)	28 ± 13	42 ± 17	24 ± 9	1.2 ± 0.3	0.9 ± 0.2	1.0 ± 0.2	56 ± 16	62 ± 18	89 ± 93
5 (F, 31)	19 ± 8	38 ± 10	21 ± 8	1.1 ± 0.2	0.9 ± 0.1	0.9 ± 0.1	57 ± 16	59 ± 13	82 ± 73
6 (M, 64)	14 ± 8	35 ± 16	14 ± 7	1.6 ± 0.4	1.0 ± 0.2	1.2 ± 0.3	53 ± 10	60 ± 15	65 ± 38
7 (F, 66)	19 ± 10	34 ± 11	19 ± 8	1.1 ± 0.2	0.9 ± 0.2	1.0 ± 0.2	72 ± 54	68 ± 34	84 ± 97
8 (F, 33)	21 ± 10	43 ± 16	19 ± 7	1.1 ± 0.2	0.9 ± 0.1	1.1 ± 0.2	56 ± 21	61 ± 16	86 ± 81
9 (F, 55)	23 ± 16	41 ± 18	22 ± 8	1.3 ± 0.4	1.0 ± 0.1	1.1 ± 0.2	60 ± 49	60 ± 16	88 ± 87
10 (F, 24)	29 ± 19	46 ± 15	25 ± 10	1.1 ± 0.2	0.9 ± 0.1	1.0 ± 0.2	56 ± 13	51 ± 4	73 ± 69
11 (M, 48)	7 ± 7	42 ± 15	19 ± 8	1.5 ± 0.8	1.1 ± 0.3	1.2 ± 0.3	57 ± 12	62 ± 18	85 ± 100
12 (M,25)	53 ± 16	59 ± 19	29 ± 11	1.1 ± 0.2	0.9 ± 0.1	1.0 ± 0.2	56 ± 9	65 ± 17	81 ± 49
Mean	25	41	21	1.2	1.0	1.1	60	60	81

## Data Availability

As per our research agreement with Philips Healthcare and our IRB regulations, the data and code used in this study are confidential but may be provided upon request when signing a data use agreement.
