# Supplementary material for: Ultra-long-TE arterial spin labeling reveals rapid and brain-wide blood-to-CSF water transport in humans
Source: Neuroimage. Author manuscript; Available in PMC 2022 Jun 30. (PMC7612938; doi:10.1016/j.neuroimage.2021.118755)
Supplement: Supplementary Material [file EMS146253-supplement-Supplementary_Material.docx]

$${}{}{}^{{}}{}{}^{{}}{}{}^{{}}$$

$${}\left( \right){}\int{}\left( {} \right){}\left( {} \right){}\left( {} \right){}$$

${}\left( \right)\left\{ \begin{aligned} \\ {}^{{}/{{}}} \\ \end{aligned} \right.$

$${}\left( \right){}^{{}/{{}}}$$

$${}\left( \right){}\int{}\left( {} \right){}\left( {} \right){}\left( {} \right)$$

$${}\left( \right){}\int{}\left( {} \right){}\left( {} \right){}\left( {} \right){}\left( \right){}$$

$${}\left( \right)\left\{ \begin{aligned} \\ {}^{{}/{{}}}{}^{{}/{{}}}\frac{{}\left( \right){}\left( \right)}{{}} \left( \right) \\ \end{aligned} \right.$$

$${}_{\left( \right)}\left( \right){}^{{}/{{}}}$$

$${}{}$$

$${}\left( \right){}{}{}^{{}/{{}}}\left( {}^{{}/{{}}} \right)$$

$${}\left( \right){}{}^{{}/{{}}}\left[ {}\left( {}^{\left( \right)/{{}}} \right){}{}^{{}/{{}}} \right]$$

$${}\left( \right){}{}{}^{{}/{{}}}{}^{\left( \right)/{{}}}\left( {}^{{}/{{}}} \right)$$

$${}\left( \right){}{}^{{}/{{}}}\left[ {}{}^{\left( \right)/{{}}}\left( {}^{{}/{{}}} \right){}{}^{\left( \right)/{{}}}\left( {}^{{}/{{}}} \right) \right]$$

$$\frac{}{{}}\frac{}{{}}\frac{}{{}}$$

$$\frac{}{{}}\frac{}{{}}\frac{}{{}}$$

$$\frac{}{{}}\frac{}{{}}\frac{}{{}}$$

$$\frac{}{{}}\frac{}{{}}\frac{}{{}}$$

$${}\left( \right){}\left( \right)$$

$${}\left( \right){}{}{}^{{}/{{}}}{}^{{}/{{}}}\left( {}^{{}/{{}}} \right)$$

$${}\left( \right){}{}^{{}/{{}}}{}^{{}/{{}}}\left[ {}\left( {}^{\left( \right)/{{}}} \right){}{}^{\left( \right)/{{}}} \right]$$

$${}\left( \right){}{}{}^{{}/{{}}}{}^{{}/{{}}}{}^{\left( \right)/{{}}}\left( {}^{{}/{{}}} \right)$$

$${}\left( \right){}{}^{{}/{{}}}{}^{{}/{{}}}\left[ {}{}^{\left( \right)/{{}}}\left( {}^{{}/{{}}} \right){}{}^{\left( \right)/{{}}}{}^{{}/{{}}} \right]$$

$${}\left( \right){}\left( \right){}^{{}/{{}}}{}{}{}^{{}/{{}}}{}^{{}/{{}}}\left( {}^{{}/{{}}} \right)$$

$${}\left( \right){}\left( \right)\left( {}^{{}/{{}}} \right){}^{{}/{{}}}{}\left( \right){}^{{}/{{}}}{}{}^{{}/{{}}}{}^{{}/{{}}}\left[ {}\left( {}^{{}/{{}}} \right){}{}^{{}/{{}}} \right]$$

$${}\left( \right){}\left( \right){}^{{}/{{}}}{}{}{}^{{}/{{}}}{}^{{}/{{}}}{}^{{}/{{}}}\left( {}^{\left( \right)/{{}}} \right)$$

$${}\left( \right){}\left( \right)\left( {}^{{}/{{}}} \right){}^{{}/{{}}}{}\left( \right){}^{{}/{{}}}{}{}^{{}/{{}}}{}^{{}/{{}}}\left[ {}{}^{{}/{{}}}\left( {}^{\left( \right)/{{}}} \right){}{}^{{}/{{}}}{}^{\left( \right)/{{}}} \right]$$

$${}\left( \right){}{}^{{}/{{}}}$$

$${}\left( \right){}\left( \right)\left( {}^{{}/{{}}} \right){}^{{}/{{}}}{}{}^{{}/{{}}}$$

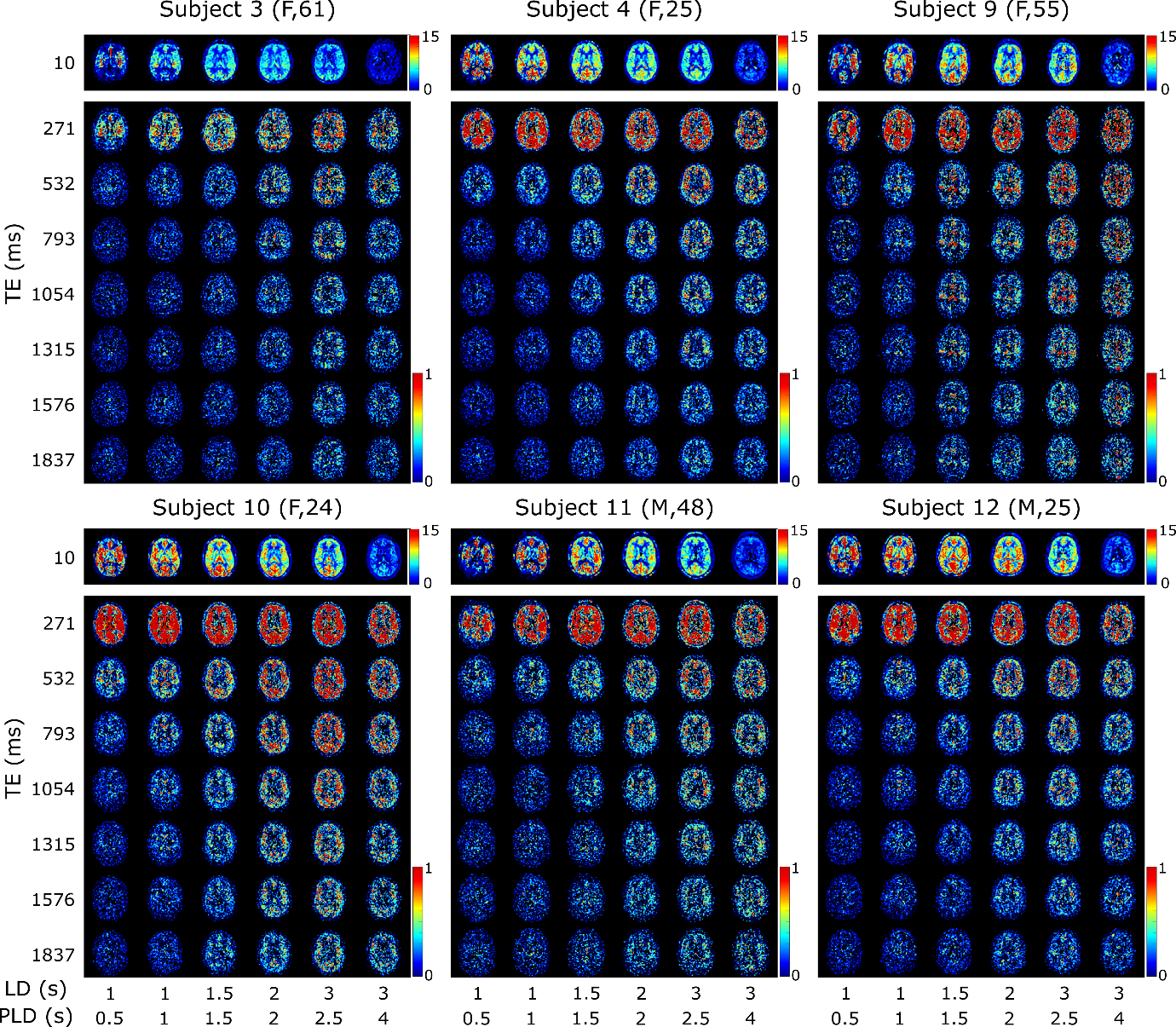


Supplemental figure S1: ASL signal for a single central slice at all TEs and PLDs for six subjects. These subjects were chosen arbitrarily to represent a range of age and sex.


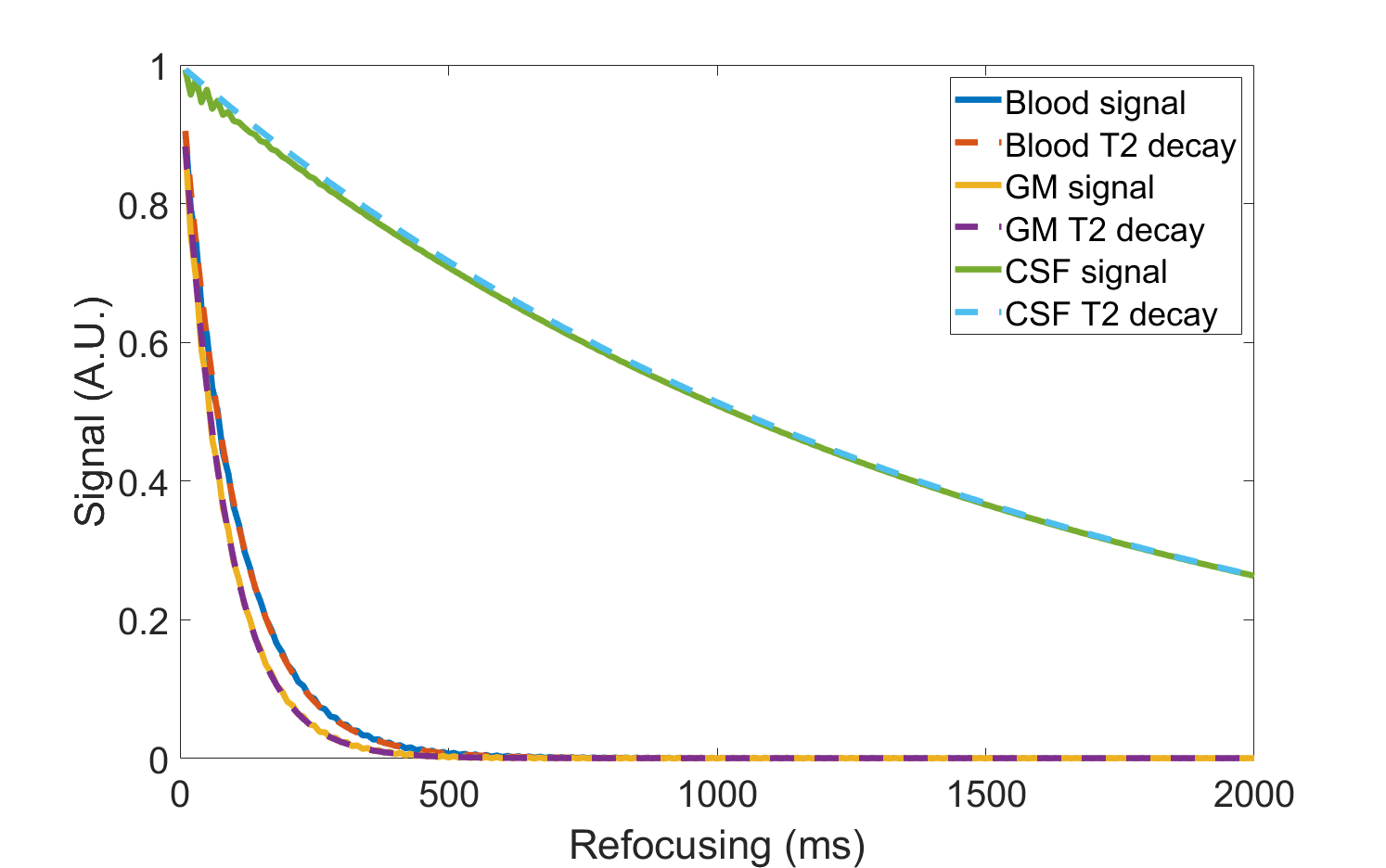


Supplemental figure S2: EPG-based simulation of the signal evolution through the (200) refocusing pulses used in this study, for blood, gray matter and CSF (dashed lines) compared to their pure T_2_ decay (solid lines).

Supplemental figure S3 (video file): Reconstructed blood (+ GM) and CSF fractions for a label duration of 3 s and PLDs from 0-5 s. This simulates more intuitively the passage of a bolus of labeled water from its arrival in the vasculature to its deposition in the tissue or CSF.
