# Supplementary material for: Ultra-long-TE arterial spin labeling reveals rapid and brain-wide blood-to-CSF water transport in humans
Source: Neuroimage. Author manuscript; Available in PMC 2022 Jun 30. (PMC7612938; doi:10.1016/j.neuroimage.2021.118755)
Supplement: Appendix [file EMS146253-supplement-Appendix.pdf]

Supplementary materials

Supplementary material associated with this article can be found, in the online version, at doi:10.1016/j.neuroimage.2021.118755.

Appendix – Solution to Model Equations

The evolution of the ASL signal must be split into two parts, before (S_1) and after (S_2) the excitation pulse, to account for the different re-

laxation times in these two regimes. In the first regime, the equations are split into three parts, i.e. before, during, and after the arrival of the bolus of labeled signal in the voxel. For S_2, not only is the time at excitation relevant, but also the echo time. To take this into account, three cases are outlined again depending on the passage of the bolus at excitation, and each case is split into parts to account for the fact that the bolus is still passing through during the readout.

Before excitation:

For $t \leq \Delta$:

$$S_{bl} = S_{CSF} = 0 \tag{A1}$$

For $\Delta \leq t \leq \Delta \pm \tau$:

$$S_{1bl}(t) = 2\alpha M_0 f T'_{1bl} e^{-\Delta/T_{1bl}} \left(1 - e^{-(t-\Delta)/T'_{1bl}}\right) \tag{A2}$$

$$\begin{aligned} S_{1CSF}(t) = {} & 2\alpha M_0 f e^{-\Delta/T_{1bl}} \Big[T_{1CSF}\big(1 - e^{-(t-\Delta)/T_{1CSF}}\big) \\ & - T'_{1CSF}\Big(1 - e^{-(t-\Delta)/T'_{1CSF}}\Big)\Big] \end{aligned} \tag{A3}$$

For $\Delta \pm \tau \leq$ t:

$$S_{1bl}(t) = 2\alpha M_0 f T'_{1bl} e^{-\Delta/T_{1bl}} e^{-(t-\Delta)/T'_{1bl}} \left(e^{\tau/T'_{1bl}} - 1\right) \tag{A4}$$

$$\begin{aligned} S_{1CSF}(t) = {} & 2\alpha M_0 f e^{-\Delta/T_{1bl}} \Big[T_{1CSF} e^{-(t-\Delta)/T_{1CSF}} \big(e^{\tau/T_{1CSF}} - 1\big) \\ & - T'_{1CSF} e^{-(t-\Delta)/T'_{1CSF}} \Big(e^{\tau/T'_{1CSF}} - 1\Big)\Big] \end{aligned} \tag{A5}$$

$$\frac{1}{T'_{1bl}} = \frac{1}{T_{1bl}} + \frac{1}{T_{bl\to CSF}} \tag{A6}$$

$$\frac{1}{T'_{1CSF}} = \frac{1}{T_{1CSF}} + \frac{1}{T_{bl\to CSF}} \tag{A7}$$

After excitation:

For this section,

$$t = \theta + TE \tag{A8}$$

$$\theta = \tau + w \tag{A9}$$

$$\frac{1}{T'_{2bl}} = \frac{1}{T_{2bl}} + \frac{1}{T_{bl\to CSF}} \tag{A10}$$

$$\frac{1}{T'_{2CSF}} = \frac{1}{T_{2CSF}} + \frac{1}{T_{bl\to CSF}} \tag{A11}$$

3 cases are based on time at excitation time θ.

Case 1: $\theta < \Delta$

For TE $\leq \Delta - \theta$:

$$S_{2bl}(TE) = S_{2CSF}(TE) = 0 \tag{A12}$$

For $\Delta - \theta \leq$ TE $\leq \Delta \pm \tau - \theta$:

$$S_{2bl}(TE) = 2\alpha M_0 f T'_{2bl} e^{-\Delta/T_{1bl}} e^{-TE/T_{2bl}} \left(1 - e^{-(TE-\Delta+\theta)/T'_{2bl}}\right) \tag{A13}$$

$$\begin{aligned} S_{2CSF}(TE) = {} & 2\alpha M_0 f e^{-\Delta/T_{1bl}} e^{-TE/T_{2bl}} \Big[T_{2CSF}\big(1 - e^{-(TE-\Delta+\theta)/T_{2CSF}}\big) \\ & - T'_{2CSF}\Big(1 - e^{-(TE-\Delta+\theta)/T'_{2CSF}}\Big)\Big] \end{aligned} \tag{A14}$$

For $\Delta \pm \tau - \theta \leq$ TE:

$$S_{2bl}(TE) = 2\alpha M_0 f T'_{2bl} e^{-\Delta/T_{1bl}} e^{-TE/T_{2bl}} e^{-(TE-\Delta+\theta)/T'_{2bl}} \left(e^{\tau/T'_{2bl}} - 1\right) \tag{A15}$$

$$\begin{aligned} S_{2CSF}(TE) = {} & 2\alpha M_0 f e^{-\Delta/T_{1bl}} e^{-TE/T_{2bl}} \big[T_{2CSF} e^{-(TE-\Delta+\theta)/T_{2CSF}} \big(e^{\tau/T_{2CSF}} - 1\big) \\ & - T'_{2CSF} e^{-(TE-\Delta+\theta)/T'_{2CSF}} \big(e^{\tau/T'_{2CSF}} - 1\big)\big] \end{aligned} \tag{A16}$$

Case 2: $\Delta \leq \theta < \delta + \tau$

For $0 \leq$ TE $\leq \Delta \pm \tau - \theta$:

$$S_{2bl}(TE) = S_{1bl}(\theta) e^{-TE/T'_{2bl}} + 2\alpha M_0 f T'_{2bl} e^{-\Delta/T_{1bl}} e^{-TE/T_{2bl}} \left(1 - e^{-TE/T'_{2bl}}\right) \tag{A17}$$

$$\begin{aligned} S_{2CSF}(TE) = {} & S_{1bl}(\theta)\big(1 - e^{-TE/T_{bl\to CSF}}\big) e^{-TE/T_{2CSF}} + S_{1CSF}(\theta) e^{-TE/T_{2CSF}} \\ & + 2\alpha M_0 f e^{-\Delta/T_{1bl}} e^{-TE/T_{2bl}} \Big[T_{2CSF}\big(1 - e^{-TE/T_{2CSF}}\big) \\ & - T'_{2CSF}\Big(1 - e^{-TE/T'_{2CSF}}\Big)\Big] \end{aligned} \tag{A18}$$

For $\Delta \pm \tau - \theta \leq$ TE:

$$\begin{aligned} S_{2bl}(TE) = {} & S_{1bl}(\theta) e^{-TE/T'_{2bl}} + 2\alpha M_0 f T'_{2bl} e^{-\Delta/T_{1bl}} e^{-TE/T_{2bl}} e^{-TE/T'_{2bl}} \\ & \times \left(e^{(\Delta+\tau-\theta)/T'_{2bl}} - 1\right) \end{aligned} \tag{A19}$$

$$\begin{aligned} S_{2CSF}(TE) = {} & S_{1bl}(\theta)\big(1 - e^{-TE/T_{bl\to CSF}}\big) e^{-TE/T_{2CSF}} + S_{1CSF}(\theta) e^{-TE/T_{2CSF}} \\ & + 2\alpha M_0 f e^{-\Delta/T_{1bl}} e^{-TE/T_{2bl}} \Big[T_{2CSF} e^{-TE/T_{2CSF}} \big(e^{(\Delta+\tau-\theta)/T_{2CSF}} - 1\big) \\ & - T'_{2CSF} e^{-TE/T'_{2CSF}} \Big(e^{(\Delta+\tau-\theta)/T'_{2CSF}} - 1\Big)\Big] \end{aligned} \tag{A20}$$

Case 3: $\delta + \tau \leq \theta$

$$S_{2bl}(TE) = S_{1bl}(\theta) e^{-TE/T'_{2bl}} \tag{A21}$$

$$S_{2CSF}(TE) = S_{1bl}(\theta)\big(1 - e^{-TE/T_{bl\to CSF}}\big) e^{-TE/T_{2CSF}} + S_{1CSF}(\theta) e^{-TE/T_{2CSF}} \tag{A22}$$
